# Physiological impacts of chronic and experimental *Plasmodium* infection on breeding-condition male songbirds

**DOI:** 10.1038/s41598-023-38438-6

**Published:** 2023-08-11

**Authors:** K. M. Talbott, E. D. Ketterson

**Affiliations:** Department of Biology, Indiana University, Biology Building 149, 1001 East 3rd St, Bloomington, IN 47405 USA

**Keywords:** Ecology, Diseases, Animal physiology, Infectious diseases

## Abstract

While *Plasmodium* parasitism is common in songbirds, its impact on avian reproduction is unclear owing to conflicting reports in the existing literature. Particularly understudied is the impact of phase of infection on variation in host reproductive physiology in wild, breeding-condition birds. However, assessing the full impact of *Plasmodium* on reproductive success in the wild can be difficult because individuals experiencing severe effects of parasitism may not enter the breeding population and may be less likely to be captured during field studies. To address these factors, we quantified metrics of health and reproductive physiology in wild-caught, breeding-condition male dark-eyed juncos (*Junco hyemalis hyemalis*) before and after experimental *Plasmodium* inoculation in a captive setting. Metrics of health and reproductive physiology included activity rate, hematocrit, scaled body mass, testosterone and sperm production. Individuals already infected at capture (i.e., chronically infected) had higher levels of hematocrit than males without chronic infections. Experimentally infected males showed a larger reduction in hematocrit and activity rate as compared to controls. However, chronic infection status did not influence the extent of metric decline. Testosterone production did not vary by treatment and most birds produced sperm following inoculation. Broadly, our results suggest that male juncos exposed to *Plasmodium* during the breeding season likely experience declines in general health, but *Plasmodium* infections do not negatively impact reproductive physiology. We conclude that physiological tradeoffs in males may favor maintenance of reproductive function despite infection.

## Introduction

Identifying and quantifying the impact of common parasites on host reproduction is an important component of avian evolutionary ecology. In wild songbirds, these impacts are often quantified as the reduction in offspring number and quality attributable to parasitism. For example, *Mycoplasma gallisepticum* bacterial infection in house finch (*Haemorhous mexicanus*) nestlings reduces growth^1^, gapeworm nematode infections in female house sparrows (*Passer domesticus*) reduce hatching success^2^, and reducing parental *Plasmodium* burden increases hatching and fledging success in blue tits (*Cyanistes caeruleus*)^3^ and house martins (*Delichon urbica*)^4^.

However, focusing on variation in reproductive output may underestimate the full cost of parasitism, as severely impacted hosts might fail at any milestone between securing a breeding territory and successfully fertilizing eggs, and such failure might prevent these individuals from being studied. For example, a host severely impacted by parasitism might experience a reduction in body condition, leading to reduced energy availability for reproductive behavior and gametogenesis, which could ultimately result in that individual failing to acquire a mate for the breeding season. To better understand parasite impacts on host reproduction, experimental infection studies investigating the physiological effects of parasitism in breeding-condition hosts are needed. An ideal model parasite for this task is *Plasmodium*, a common parasite of songbirds that is also amenable to transfection^5^.

*Plasmodium* is a blood parasite found in avian hosts throughout the world wherever mosquito vectors are distributed and is one of three haemosporidian genera associated with avian malaria^6^. Upon infection, the host enters an acute phase, marked by rapid parasite replication. Clinical signs of *Plasmodium* infection can vary widely, from lethargy to anemia, anorexia, and death^7^. Following a peak in parasite load, the host will either clear the infection or enter a chronic phase, marked by relatively low parasite replication, which can extend through the lifetime of the host^7^. *Plasmodium* prevalences of over 40% are frequently reported in wild songbird populations^3,8–10^, and chronic infections are common, as evidenced by the persistence of *Plasmodium* in individual hosts sampled over multiple years^11–13^. Importantly, these prevalence estimates do not account for birds that are too ill to be captured by live trapping^14^ or those that die from *Plasmodium* infection before they are sampled. These estimates also do not account for latent infections that have exited the blood stream^5^, and hence cannot be detected using common diagnostic methods (i.e., PCR and blood smear microscopy). Furthermore, the sensitivity of microscopy and PCR may vary by parasite load and parasite genotype, respectively, which may increase the risk of false negatives^15^. Thus, the true prevalence of *Plasmodium* might be much higher than is currently known, and the risk of *Plasmodium* exposure might be especially high in certain habitats.

Despite its high prevalence, the impact of *Plasmodium* on avian reproductive success is unclear. In some species, fewer independent young are produced by parents with chronic *Plasmodium* infections^3,16^. At the behavioral level, chronically infected males may show reduced singing^17^ or courtship behavior^18^ and may experience higher rates of paternity loss when paired with an uninfected mate^19^. While these studies suggest that *Plasmodium* reduces host reproductive success, additional studies show a neutral or even positive relationship between *Plasmodium* and host reproductive success. For example, no *Plasmodium*-related differences were detected in the number of fledged young produced by male red-winged blackbirds^20^ (*Agelaius phoeniceus*) or male Australian magpies (*Gymnorhina tibicen*)^21^. Similarly, no *Plasmodium*-related differences in nestling growth rate or fledgling size were found for male collared flycatchers (*Ficedula albicollis*)^22^, or in the number of recruited offspring sired by male reed warblers (*Acrocephalus arundinaceus*)^11^. Most surprising of all, *Plasmodium* has caused significant population declines in many endemic Hawaiian songbird populations, yet chronically infected Amakihi (*Chlorodrepanis virens*) now show higher reproductive success than uninfected parents^23^. Similarly, fledging success is higher in great tits (*Parus major*) infected with *Plasmodium*^24^.

Such heterogeneity in host impacts of *Plasmodium* is often attributed to variation in the host and parasite species involved^25,26^, as well as endogenous factors such as host genotype^27,28^, nutritional state^29^ or coinfecting parasites^30,31^. Phase of infection is another possible contributor to observed heterogeneity in impacts of *Plasmodium* in wild songbirds. It is well established that the acute phase of infection impacts hosts more severely in comparison to the chronic phase^7^. However, comparing the impacts of acute infections, chronic infections, and reinfection in chronically infected birds (here we use ‘superinfection’ in the case of reinfection with the same strain and ‘coinfection’ in the case of distinct strains) is difficult in wild hosts, as the timeline of parasite exposure is often unknown and exposure to parasite vectors may continue throughout the course of study.

Importantly, we might expect distinct physiological responses to new *Plasmodium* infections depending on whether a host is chronically infected. One hypothesis is that coinfecting strains might lead to increased virulence (e.g., through facilitation between distinct strains), as is demonstrated in laboratory mice^32,33^. Alternatively, chronically infected birds might experience little or no parasite replication upon superinfection, if both strains share similar antigens^34,35^ or if there is resource competition between strains^36^. However, these competing hypotheses have yet to be tested in wild, breeding-condition songbirds.

To investigate the physiological impacts of parasitism in breeding-condition songbirds while disentangling variation attributable to phase of infection, we employed observational and experimental approaches in a North American migratory songbird, the dark-eyed junco (*J. h. hyemalis,* hereafter ‘junco’)^37^. *Plasmodium* has been noted in many junco populations^38–41^, with prevalences up to approximately 71%^41^. Because only males of this species reliably show recrudescence in captivity in response to photoperiod stimuli (E.K., personal observation), we focused our study on male juncos.

To assess reproductive physiology, we measured GnRH-induced testosterone (‘max T’), cloacal protuberance volume (‘CP volume’), sperm count, and deformed sperm proportion. Testosterone helps support vertebrate spermatogenesis and reproductive behavior^42^. For example, max T reflects the circulating level of testosterone produced in male juncos during aggressive intrasexual encounters^43^. In male juncos, gonadal recrudescence in the early spring increases max T, and the peak in max T coincides with territory establishment^44^. Max T is also positively associated with offspring number^45^ and with the expression of while tail plumage, a trait preferred by female juncos^43^. The cloacal protuberance is an external sperm storage organ that increases in size as gonads grow internally^46^. CP volume is positively associated with fertilization success in highly promiscuous species such as tree swallows (*Tachycineta bicolor*)^47^. Although this relationship has not yet been investigated in juncos, extra-pair copulation and paternity loss is common in juncos^48^, suggesting that some level of sperm competition is likely.

We also measured three general proxies of health: scaled body mass (‘body index’), hematocrit, and activity rate. Mass scaled by tarsus length accounts for variation in body frame size when comparing weight among individuals^49^; thus, individuals with relatively higher body index values have larger fat stores and/or lean muscle. Mass loss occurs in some species experimentally inoculated *Plasmodium*^34,50,51^ (although not all^52,53^), and is positively correlated with likelihood of mortality^50,51^. Hematocrit is quantified as the proportion of packed erythrocytes in a whole blood sample, and hematocrit decline is one of the most common clinical symptoms of *Plasmodium* infection, as parasite replication bursts host erythrocytes^5^; hematocrit is also sensitive to other infections, dehydration, and nutrient deficiencies^54^. Hematocrit tends to increase in male juncos and other North American songbirds prior to spring migration and decrease slightly throughout the breeding season^55,56^. Importantly, hematocrit is thought to reflect aerobic capacity^57^, which might be important in supporting the reproductive behavior of male songbirds. Reduced activity is commonly reported in birds experimentally inoculated with *Plasmodium*^14,50^ and is thought to benefit the host by reducing nutrient and energy expenditure, thereby increasing resources available for immune function^58^. While activity rate cannot be directly translated to fitness, a reduction in activity might correlate with reduced foraging^58^ as well as reduced courtship behavior^18^ and territoriality.

Using these metrics of reproductive physiology and health, we tested three hypotheses in wild-caught, breeding-condition male juncos. First, anticipating that some subjects would already be carrying long-term (i.e., chronic) infections upon capture, we tested the hypothesis (1) that chronically infected males would be of higher quality than uninfected individuals due to survivor bias. If this hypothesis were supported, we predicted that chronically infected males would show higher values in metrics of overall health and reproductive physiology than those entering the study without chronic infections. If this prediction were borne out, it would also suggest that carrying chronic infections might not be detrimental to male reproductive success, but rather be a correlate of higher quality.

Second, we tested the hypothesis (2) that new *Plasmodium* infection (i.e., ‘acute’, simulated by experimental inoculation) would negatively impact overall health and reproductive physiology in breeding-condition males. We predicted that experimentally inoculated males would show larger declines in metrics of health and reproductive physiology from baseline to post-inoculation, as compared to controls. Third, we tested the hypothesis (3) that males with and without chronic *Plasmodium* infections would respond differently to acute infection, simulated by experimental inoculation. We predicted that males with chronic infections would outperform males without chronic infections in metrics of health and reproductive physiology following *Plasmodium* inoculation. If this prediction were supported, it would suggest that males that survive their first *Plasmodium* infection and live with chronic infections might exhibit higher reproductive success than those without chronic infections, especially in environments with a high risk of *Plasmodium* exposure.

## Results

### Hypothesis 1

Pre-inoculation comparisons of reproductive physiology and health indices based on presence of chronic *Plasmodium* infections.

Prior to experimental inoculation, hematocrit values were higher in chronically infected birds (0.51 ± 0.01 SE) than in those without chronic infections (0.49 ± 0.01 SE; t_20.3_ = − 2.29, *p* = 0.03). We detected a trend toward higher body condition indices in birds with chronic infections (t_16.8_ = − 1.86, *p* = 0.08). Similarly, we detected a trend toward higher cloacal protuberance (CP) volume in chronically infected birds (t_22.4_ = − 1.82, *p* = 0.08). However, gonadotropin-releasing hormone induced maximum testosterone (‘max T’) concentration (W = 77, *p* = 1.00) and activity rate (t_17.99_ = 0.63, *p* = 0.54) did not vary by baseline infection status; see Table 1 for full baseline summary statistics.

**Table 1:** Differences in pre-inoculation metrics of health and reproductive physiology between male juncos with (n = 15) and without (n = 11) chronic *Plasmodium* infection.

Metric	Uninfected mean	± SE	Chronically infected mean	± SE	Difference between groups
GnRH-induced testosterone levels (ng/ml)	4.46	0.53	5.12 (n = 14)	0.78	W = 77, *p* = 1
Activity rate (movements/min)	94.06 (n = 9)	5.51	89.50 (n = 14)	4.69	t = 0.63, df = 17.99, *p* = 0.54
Cloacal protuberance volume (mm^3^)	154.64	9.45	177.75	8.59	t = − 1.81, df = 22.42, *p* = 0.08
**Hematocrit**	**0.49**	**0.01**	**0.51**	**0.01**	**t = − 2.29, df = 20.31,p= 0.03** **(95% CI − 0.04 to − 0.002)**
Body index (g mass/mm tarsus length)	0.98	0.02	1.03	0.01	t = − 1.86, df = 16.80, *p* = 0.08

Sample sizes are indicated in parentheses under sample means where data are not available for all birds. Differences between groups reflect the results of t tests or Wilcoxon rank sum tests as appropriate. Significant results (i.e., *p* ≤ 0.05) are in bold and accompanied by a 95% confidence interval.

### Hypotheses 2 and 3

Costs of parasite exposure through experimental *Plasmodium* inoculation.

**Figure 1:**
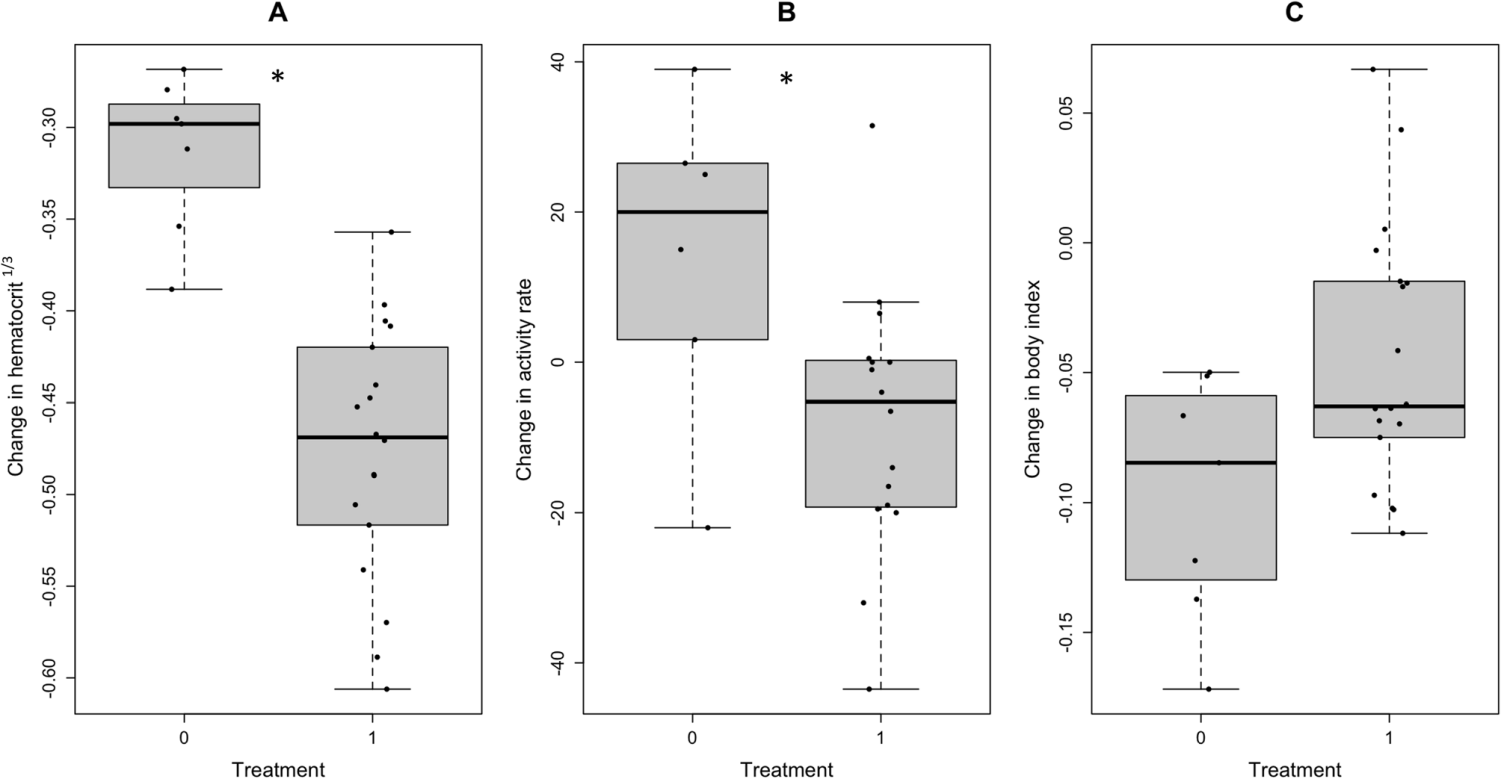
Changes in metrics of health and reproductive physiology in male dark-eyed juncos following *Plasmodium* inoculation. Treatments include males experimentally inoculated with *Plasmodium*-infected blood (1) or control blood (0). Values are given as differentials (change from baseline to post-inoculation values), using data collected on the date of lowest hematocrit for post-inoculation values; negative values indicate a decline in metric values from baseline to post-inoculation. (**A**) cube root-transformed hematocrit (proportion of packed erythrocytes in a whole blood sample) differentials, (**B**) activity rate (movements per minute) and (**C**) body condition index (mass in grams/tarsus length in mm). Dots represent individual birds, medians are represented by lines across each box, and asterisks (*) indicate significant differences between groups at α = 0.05.

A multiple linear regression investigating the effect of treatment and baseline infection status on cube root-transformed hematocrit differentials was statically significant (R^2^ = 0.62, F_2,22_ = 17.94, *p* < 0.001), and showed that experimentally inoculated birds experienced a decline in hematocrit (β = − 1.16, *p* < 0.001), while baseline infection status did not influence hematocrit differentials (β = − 0.03, *p* = 0.21); see Fig. 1. A model with the same predictors for activity rate differentials was significant (R^2^ = 0.35, F_2,19_ = 5.06, *p* = 0.02), showing a general decrease in activity for experimentally inoculated birds (β = − 21.10, *p* = 0.03), but a trend of increased activity in inoculated birds that had chronic infections at baseline (β = 13.60, *p* = 0.09). A model with the same predictors for body index differentials trended toward significance (R^2^ = 0.20, F_2,22_ = 2.82, *p* = 0.08), showing an increase in body index from baseline to post-inoculation in experimentally inoculated birds (β = 0.05, *p* = 0.03), while baseline infection status had no effect on body index differentials (β = − 0.01, *p* = 0.81). Models with the same predictors for CP volume differentials (R^2^ = 0.06, F_2,22_ = 0.74, *p* = 0.49), max T differentials (R^2^ = 0.10, F_2,22_ = 1.17, *p* = 0.33), sperm count at three weeks post-inoculation (R^2^ = 0.09, F_2,22_ = 1.05, *p* = 0.37) and deformed sperm proportion at three weeks post-inoculation (R^2^ = 0.04, F_2,22_ = 0.47, *p* = 0.63) were not significant. See Table 2 for full summary statistics.

**Table 2:** Changes in metrics of health and reproductive physiology following experimental *Plasmodium* inoculation. Changes were calculated by subtracting post-inoculation values from baseline values; negative values indicate a decline in value from baseline to post-inoculation. Post-inoculation data reflect measurements collected on the date of each bird’s lowest hematocrit value within 5 weeks following *Plasmodium* inoculation. Sperm count and deformed sperm proportion were assessed only at three weeks post-inoculation and summary statistics are for these measurements. Values reflect non-transformed data.

Metric	Control mean	± SE	Experimental mean	± SE
GnRH-induced testosterone (ng/ml)	− 1.96	0.86 (n = 7)	− 0.32	0.75 (n = 18)
Activity rate (movements/min)	14.42	8.79 (n = 6)	− 8.09	4.39 (n = 16)
Cloacal protuberance volume (mm^3^)	− 51.75	16.57 (n = 7)	− 39.26	6.04 (n = 18)
Hematocrit (cube root transformed)	− 0.31	0.02 (n = 7)	− 0.48	0.02 (n = 17)
Body index (g mass/mm tarsus length)	− 0.1	0.02 (n = 7)	− 0.04	0.01 (n = 18)
Sperm count*	147.69	25.71 (n = 8)	154.94	12.16 (n = 17)
Deformed sperm proportion*	0.15	0.03 (n = 8)	0.19	0.02 (n = 17)

*Summary statistics are for data collected at three weeks post-inoculation.

## Discussion

We asked whether chronic *Plasmodium* infections were associated with differences in metrics of health and reproductive physiology in breeding-condition male dark-eyed juncos. We then investigated whether experimental inoculation with *Plasmodium* influenced the same metrics, and whether these impacts varied between males with and without pre-existing, chronic *Plasmodium* infections. As predicted in our first hypothesis, we found that chronically infected males tended toward higher values of health-related metrics and one reproductive metric, including higher hematocrit, a trend toward higher body index values, and a trend toward larger CP volumes, compared to males without chronic infections. We found no differences in max T (see Fig. S2) or activity rates between males with and without chronic infections at baseline. Altogether, the results provide varied support for the hypothesis that chronically infected males are of higher quality than those without chronic infections.

As predicted in our second hypothesis, experimental males responded to *Plasmodium* inoculation with a larger decline in hematocrit compared to controls. Similarly, the experimental group showed significantly larger reductions in activity rate, and there was a trend of smaller reductions in activity among the experimental males that had chronic infections at baseline. Interestingly, and not as predicted, we noted a trend of increased body condition index in the experimental group compared to the controls. Contrary to what we predicted in hypothesis 3, we found no significant relationship between chronic infection status and changes in any of the metrics quantified. Together, these results provide important insights into the functional impacts of both chronic *Plasmodium* infections and new exposures, and generally suggest that reproductive physiology is maintained during both phases of *Plasmodium* infection in male juncos.

### Hypothesis 1

Metrics of health and reproductive physiology vary based on chronic Plasmodium infection.

Our results broadly suggest that chronic infections might not impose costs in terms of reproductive physiology or health as measured here. Conversely, chronically infected males showed higher levels of hematocrit than males without chronic infections. This is surprising, given that existing studies generally show either a negative association^59,60^ or no association between hematocrit and chronic *Plasmodium* infection in songbirds^61–63^. Although we detected no differences in max T between birds with and without chronic infections, it is possible that the chronically infected birds generally have higher levels of circulating testosterone, which is known to support hematopoiesis^64^. The trend of higher body mass in chronically infected males is difficult to interpret, as we did not assess fat scores and pectoral muscle. However, we would predict both metrics to be positively associated with reproductive function; for example, body index is positively associated with numbers of independent young in adult both male and female crimson finches (*Neochmia phaeton*)^65^. Although body index has not been tested explicitly as a predictor of junco reproductive success, body size as approximated by wing length is associated with higher reproductive success in male juncos^66^. Most interesting of all, CP volume, an estimate of sperm storage capacity, tended to be higher in chronically infected males than those without chronic infections. CP volume is predictive of fertilization success in male tree swallows (*Tachycineta bicolor*)^47^, and while this relationship has yet to be tested in juncos, it is possible that higher CP volumes in chronically infected males could increase reproductive success.

It is unclear why chronically infected males showed higher levels of hematocrit and tended to have higher CP volume and body index values. It is possible that *Plasmodium* parasitism is a sufficiently selective force, particularly in juveniles, that survivors of acute infection tend to be those with the physiological or genetic background to support these traits. Although the impact of *Plasmodium* on yearly survival of juncos is unknown, mortality has been demonstrated in naïve juvenile crossbills (*Loxia curvirostra*) experimentally inoculated with *Plasmodium elongatum*^67^.

Age is another potential explanation for our results, although we were unable to control for age in our study. Juncos are more likely to be diagnosed with chronic haemosporidian infections as they age^68^, which means the chronically infected group might have been primarily composed of males in their second year or older. Older juncos are slightly heavier than adults in their first year^69^, indicating that age could account for the trend of higher baseline body index values among the chronically infected juncos. In avian species, males show a slight increase^70^ or decrease in hematocrit as individuals mature beyond their first year^71^; however, no relationship between age and hematocrit was detected in a close relative of the junco, the white-crowned sparrow (*Zonotrichia leucophrys*)^72^. Similarly, the relationship between CP volume and age is unclear in juncos, with some studies showing smaller testes and CP volumes in adults in their first year, compared to those in their second year and older^73,74^, while another study showed no relationship between CP volume and age^75^. Another possible explanation is that chronic infection increases CP volume through terminal investment, if the infection reduces an individual’s likelihood of future reproductive opportunities^24,76^. The likelihood of this possibility is unclear, as the impact of chronic infections on junco survivorship has not yet been tested. Chronic infections are associated with shortened lifespan in some songbirds, such as great reed warblers (*Acrocephalus arundinaceus*)^12^ and great tits (*Parus major*)^24^, but not in others^11,77^.

It is plausible that age explains the higher hematocrit and trends of higher body index values and CP volume that were detected in the chronically infected group, compared to the group without chronic infections. However, it is important to note that the latter group may include not only uninfected males, but also those with undetectable infections, as well as individuals who have recovered from past infection. Thus, additional study is needed to clarify the interactive effect of age and chronic *Plasmodium* infection on junco physiology. In addition, further research is needed to determine whether hematocrit, body index, and CP volume vary among individuals with undetectable *Plasmodium* infections, those that recover from it, and those that avoid infection altogether.

### Hypotheses 2 and 3

Response to experimental *Plasmodium* inoculation.

Over the course of the experiment, all males showed a decrease in hematocrit; this corresponds with the gradual decline in hematocrit commonly seen between spring migration and fall molt in many songbirds^54,78,79^. However, the decline in hematocrit, as well as activity rate, was larger in the *Plasmodium*-inoculated group. Similar results were seen in other experimental infection studies of songbirds^14,50,52^, although ours is the first to our knowledge to use breeding-condition birds. Minimum hematocrit values for five birds fell below 35%, the threshold for avian anemia^80^. We would expect this to have serious negative consequences for overall health, yet no birds in the experimental group showed any other clinical signs of severe illness (e.g., emaciation, lethargy, or neurologic symptoms). The reasons for this are unclear, although the impact of *Plasmodium* infection is known to vary by host species, even among passerines that have co-evolved with *Plasmodium*^52^. Other possible factors might include a relatively small dose of inoculum, a relatively non-virulent strain of *Plasmodium*, or favorable conditions of captivity; captive conditions include controlled climate, an absence of predators, and perhaps most importantly, adequate nutrition. For example, several studies have found no effect of *Plasmodium* inoculation on songbird mass, contrary to predictions; this is thought to be attributable to ad libitum access to food and reduced activity in laboratory conditions^53,67,81^. Given the trend of increased body index values in the experimental group, it is likely that our *Plasmodium*-inoculated birds consumed food at a slightly higher rate than the control group, and this may have reduced the impact of infection. Although responses to the experimental infection might be interpreted as subtle, we would expect anemic birds to show more serious clinical signs in the wild, where they would experience nutritional and thermoregulatory challenges.

Following inoculation, we found no effect of treatment or chronic infection on sperm count or deformed sperm proportion. This is a surprising result, given that laboratory mice experimentally infected with *Plasmodium* show declines in sperm count and other indices of semen quality^82,83^. In addition, male Amakihi naturally infected with *Plasmodium* show a trend of higher circulating levels of testosterone compared to uninfected males^84^. Conversely, Western fence lizards (*Sceloporus occidentalis*) naturally infected with *Plasmodium* have smaller testes than uninfected lizards^85^. Our results could indicate that the dose used in our experiment was insufficient to induce declines in sperm count or that the *Plasmodium* strain in our study is not highly virulent; conversely, it is also possible that sperm production is prioritized in breeding-condition birds experiencing parasitism. Further work is needed to determine if infection intensity is associated with trade-offs between sperm production and self-maintenance, and whether *Plasmodium* influences other metrics of semen quality in songbirds.

Interestingly, we also detected no differences in max T between males with and without chronic infections, nor did we find an effect of *Plasmodium* inoculation on max T (see see Fig. S2). Although the literature is mixed (reviewed in^86^), testosterone is generally thought to be immunosuppressive^87^, which would indicate that individuals must either lower testosterone levels in response to parasitism or incur some cost to maintaining normal levels of testosterone. Research on testosterone dynamics in relation to *Plasmodium* is limited, but existing studies show that naturally infected human men^88^ and Western fence lizards^85^ show lower levels of circulating testosterone, as do laboratory mice following experimental infection^82^. However, our results do not align with these findings. This may indicate that testosterone levels are not influenced by chronic or acute *Plasmodium* infection in this host species. Alternatively, this unexpected result might be attributable to our methods, as we quantified maximum levels of testosterone following a GnRH challenge, while the previously mentioned studies quantified natural, circulating levels of testosterone. When examining the relationship between testosterone and reproductive traits, max T is often more likely to be associated with the trait of interest than are circulating levels, which vary throughout the day (see discussion in^89^). However, because circulating testosterone levels tend to be lower than max T levels for most of the day^89^, and the bloodstream is the interface between parasite and host, additional work investigating the impact of chronic and experimental *Plasmodium* infection on circulating T is warranted.

Contrary to our third hypothesis, we found no impact of chronic infection on the extent of metric decline in juncos following *Plasmodium* inoculation. Previous experimental infection studies have shown substantial variation in the impact of *Plasmodium* coinfection on host health, based on the species involved^32–36^. Our results might be attributable to individual-level variation within the chronically infected group, based on variation in identity of the chronically infecting strains. Conversely, it is possible that the inoculating strain is sufficiently virulent to mask the effect of the chronically infecting strains. This was seen in a similar study in which common crossbills (*Loxia curvirostra*) experimentally inoculated with both *P. elongatum* and *P. relictum* experienced the same decline in hematocrit as crossbills inoculated with *P. elongatum* only; however, distinct mechanisms contributed to these apparently similar declines in hematocrit^26^. Thus, chronic infection may have influenced other physiological metrics in the experimental group that were not quantified in this study.

Importantly, female juncos were not included in this study because they cannot be brought into full breeding condition reliably in captivity, whereas male gonadal development is induced by increasing photoperiod^90^. Additional work in a host species where both sexes are amenable to captive breeding is needed. This is particularly important considering the drop in hematocrit female songbirds experience prior to egg laying^91^, which might influence sex-specific trade-offs between reproductive and immune function. Further investigation would provide important insights on the relative impacts of chronic infections, acute infections, superinfections, and coinfections on each sex, which is required to understand the full impact of *Plasmodium* on host reproductive success.

### Conclusions

Altogether, our results provide modest support for the hypothesis that *Plasmodium* selects for higher quality males in terms of higher hematocrit, although additional work is needed to determine whether this result is attributable to quality per se or simply to age. Our hypothesis that acute infection reduces metrics of health and reproductive physiology was partially supported: while the experimental group showed larger reductions in hematocrit and activity rate decline, we did not detect any impacts of experimental infection on reproductive physiology (max T, CP volume, sperm count, or deformed sperm proportion). This suggests that reproductive function may be prioritized over self-maintenance at the physiological level in male juncos. Finally, we hypothesized that male juncos would respond differently to *Plasmodium* inoculation depending on whether they also carried chronic infections; however, this hypothesis was not supported. This suggests that male juncos acquiring *Plasmodium* infection during the breeding season are likely to experience similar health impacts regardless of whether they already carry chronic infections.

Broadly, this study suggests that both chronic and acute *Plasmodium* infections may not negatively impact reproductive physiology of male juncos. However, it is unclear whether acutely infected males that prioritize reproductive physiology over self-maintenance experience survival costs. Future studies addressing this question might also incorporate different *Plasmodium* strains and more ecologically relevant levels of food availability to determine whether our results are replicable in the wild. Finally, additional work is also needed to address the impact of *Plasmodium* on female songbird reproductive physiology, as physiological tradeoffs between self-maintenance and reproduction may vary between males and females facing parasitism during the breeding season.

## Methods

### Bird capture and care

Juncos are small Passerellid songbirds distributed throughout North America; the migratory subspecies *J. h. hyemalis* breeds at higher latitudes and overwinters at lower latitudes^37^. For the first three weeks of March of 2020, we captured migratory juncos using corn and millet-baited mist nets near Bloomington, Indiana. Because female juncos do not enter breeding condition in captivity as reliably as males, we used only males for this project. We determined sex based on plumage^37^ and confirmed sex in ambiguous individuals by using PCR with primers P2/P8^92^ (see Supplement for full PCR details).

Males were maintained in an air-conditioned (approximately 65 °F) indoor aviary near Bloomington, Indiana for the duration of the experiment (Kent Farm Biological Observatory). Birds were given water and food ad libitum; feed consisted of seeds (millet and shelled sunflower seeds) and a high protein mixture (puppy chow, boiled egg, shredded carrot, and avian vitamin supplement). From the time of capture until the third week of March, free-flying birds were housed in a single large room (18 ft 7 in × 18 ft) with light schedules shifted weekly to reflect the local photoperiod in Bloomington, Indiana. From the third week of March until the third week of May, we held birds at 12:12L (HH:MM of light) to slow gonadal development until we could proceed with the experiment. This was done due to possible restrictions on live animal research that were pending at the time in response to the COVID-19 pandemic. On the third week of May (5 weeks before inoculation), we advanced the photoperiod to 14:00L to simulate migration to breeding grounds at higher latitudes. For the following 10 weeks, we increased the photoperiod by 10 min each week to simulate the progression of the breeding season. Three weeks before experimental inoculation with *Plasmodium*, we housed males in individual cages, with eight to ten cages each in three rooms (total n = 26). See Fig. S1 in the supplement for a diagram of experimental design.

### Diagnostics and experimental inoculation

To identify a *Plasmodium* strain for this experiment, we screened blood samples of wild-caught juncos housed for other projects at Kent Farm Biological Observatory. We used Maxwell RSC Whole Blood DNA kit (Promega Corporation, Madison, WI, USA) to extract DNA from whole blood samples and used a nested PCR protocol^93^ (see Supplement for full PCR details) to screen extracted DNA for the presence of haemosporidian parasites. For samples testing positive for *Plasmodium/Haemoproteus*, we used a QIAquick gel extraction kit (Qiagen, Hilden, Germany) to purify PCR product, which we submitted for Sanger sequencing. We did not screen for *Leucocytozoon*, as this parasite cannot be transmitted experimentally without an arthropod vector^5^. We manually trimmed sequences of non-coding data and used the NCBI Blastn tool for parasite identification, and deposited the sequence of the selected strain at NCBI (OP807039). This strain, tentatively identified as *P. polare,* was passaged (i.e., inoculated into a naive host) once for pilot work before we amplified it for this study. We amplified the parasite by experimentally inoculating three juncos (i.e., ‘amplifiers’) that were PCR negative for *Plasmodium/Haemoproteus* when screened using the protocol described above. Amplifiers received an intramuscular injection of 87.5 μL of fresh donor blood and sodium citrate in a 4:1 ratio.

Immediately before inoculating birds for this project, we collected small blood samples (< 75 μL) from each of the 26 newly captured males to test for the presence of chronic *Plasmodium* infections. We used PCR to screen extracted DNA, using primers L9 and NewR^3^ (see Supplement for full PCR details). Results showed 15 males with and 11 males without chronic *Plasmodium* infections. For simplicity, PCR-negative males will be referred to as ‘birds without chronic infections’; however, it should be noted that PCR-negative males could have cleared *Plasmodium* infections in the past or be carrying latent infections.

Three weeks after infecting amplifiers, we collected blood smears from each amplifier to confirm *Plasmodium* infection. We scanned blood smears for five minutes each under a light microscope, using oil immersion at 100×magnification; we confirmed that all amplifiers were producing infected erythrocytes. We then performed experimental inoculations on the 26 males captured for this project. Eighteen of these birds (‘experimental group’) received a 40 μL injection of fresh amplifier blood and sodium citrate in the ratio described above; eight birds (‘control group’) received a 40 μL injection of fresh blood from PCR-negative juncos and sodium citrate in the same ratio. We pooled infected and uninfected blood respectively and mixed each pool with the appropriate volume of sodium citrate. Injections were then prepared from these pools using insulin syringes and were administered in the pectoral muscle. Inoculations took place over three days, with one room of birds inoculated per day. To standardize the composition of each inoculum, the same ratio of blood from each individual amplifier was mixed each day; three amplifiers and the original donor were bled to prepare the *Plasmodium* inoculum and two uninfected donors were bled to prepare the control inoculum. We confirmed successful infection of *Plasmodium*-inoculated birds at three weeks post-inoculation using the microscopy protocol described above.

### Data and sample collection

We collected pre-inoculation (‘baseline’) data during the week prior to experimental *Plasmodium* inoculation; post-inoculation, we sampled once per week over five weeks. Data were collected over three days for each sampling session, with one room sampled per day (see Fig. S1 for a diagram of experimental timeline). The order of room sampling was held constant across the project to ensure all birds were sampled exactly once per week. For pre-inoculation and post-inoculation sessions, we collected measures of activity rate, hematocrit, and CP volume. We assessed activity rate by counting the number of hops and/or flight movements in a bird’s cage over the course of one minute. We then removed birds from their cages and measured body mass using a digital scale to 0.01 g (Mettler-Toledo, Columbus, OH, USA), which we converted to a scaled body condition index (‘body index’) by dividing mass (g) by tarsus length (mm). We measured cloacal protuberance length and diameter using calipers to 0.01 mm (SPI, Maplewood, NJ, USA); these data were converted to volume by calculating the volume of a cylinder^94^.

We collected a small blood sample (< 70 µL) for measuring hematocrit by pricking the brachial vein and collecting blood in a heparinized microcapillary tube. Blood samples were held on ice packs until daily sample collection was complete (~ three h). Microcapillary tubes were centrifuged at 10,000 rpm for five minutes, after which we used a ruler to quantify packed erythrocytes relative to the entire blood sample to calculate hematocrit. Pre-inoculation body index, hematocrit, and CP volume data represent the mean of two measurements taken within one week prior to inoculation. We collected all blood samples during the same three-hour window (beginning approximately two h after lights turned on) to reduce possible diel variation in hematocrit attributable to hydration state.

During the week of inoculation and again at three weeks post-inoculation, we administered gonadotropin-releasing hormone (‘GnRH’) injections to assay maximum testosterone levels (‘max T’) as described elsewhere^44^. We selected three weeks post-inoculation for the GnRH injection to quantify max T during the time when we predicted that experimental birds would be experiencing the severest impacts of parasite replication, as peak *Plasmodium* loads occur slightly more than two weeks after inoculation in a related species, the song sparrow (*Melospiza melodia*)^95^. Each bird received an intramuscular injection of 1.25 µg of chicken GnRH (Bachem Americas Inc., Torrance, CA) in 50 µL of 0.1 M phosphate-buffered saline solution. Thirty minutes after injection, we collected a blood sample (~ 50 µL) for quantification of max T. Blood samples were held on ice packs until daily sample collection was complete (~ three h) We centrifuged blood samples at 10,000 rpm for five minutes and pipetted off the plasma, which was stored at − 20 °C until hormone extraction (September 2021). We extracted hormones from 20 μL plasma aliquots with diethyl ether three times and dried samples with nitrogen gas; for plasma samples < 20 μL (12 samples), we added molecular grade water to bring the total volume to 20 μL, and then corrected for added water when calculating testosterone concentrations. We quantified testosterone using a commercial enzyme immunoassay kit (Enzo Life Sciences ADI-900-065, Farmingdale, NY, USA) according to manufacturer’s instructions and as described elsewhere^96^. Two samples with high percentage bound (> 80%) were assigned a value equal to half (0.31 ng/μL) of the lowest observed value (0.62 ng/μL). Intraplate variation was 4.9% for plate one, 1.8% for plate two, and 9.0% for plate three; interplate variation was 1.7% between plates one and two, and 5.5% between plates two and three.

At three weeks post-inoculation we also quantified sperm. Following cloacal massage^97^, we collected sperm samples in non-heparinized glass hematocrit tubes and used calipers to measure ejaculate size (mm) in the tube. We then diluted samples 101% using either Lago avian semen extender (Hygieia Biological Laboratories, Woodland CA, USA) or phosphate buffered saline, based on availability. We used a hemocytometer to count the total spermatozoa in two 10 μL aliquots for each sample as described elsewhere^98^, and then calculated the mean of the two counts. While counting sperm, we also noted the number of malformed spermatozoa within each count (i.e., those with malformed heads), and calculated the proportion of deformed sperm within each total sperm count.

### Statistical analysis

We applied Welch’s T-tests and Wilcoxon rank-sum tests to the pre-inoculation data as appropriate to determine whether metrics of health and reproductive physiology varied between males with and without chronic *Plasmodium* infections. To identify the sampling period associated with the greatest physiological impact of parasite replication, we used hematocrit as a proxy for parasite load and identified the post-inoculation sampling point with the lowest hematocrit for each bird. Using data collected on the date of lowest hematocrit (body index, hematocrit, CP volume, and activity rate), we then calculated individual changes in each metric from baseline to date of lowest hematocrit (‘differentials’). We then used multiple linear regression to determine whether treatment (control or experimental) or baseline infection status (chronically infected or uninfected) predicted differentials for each metric. We used a cube root transformation to normalize hematocrit differential data. To calculate max T differentials, we compared baseline data and data collected at three weeks post-inoculation for all birds, as max T was measured only on those dates. We used a cube root transformation to normalize max T differential data. Because sperm count and deformed sperm proportion were quantified only at three weeks post-inoculation, we used multiple linear regression to investigate the impact of baseline infection and treatment on week three data. For all linear regressions, we used a Shapiro–Wilk test to confirm normality of model residuals (all models *p* > 0.05).

Where necessary, we excluded data from birds that were missing certain measurements. For example, one bird showed consistently low activity rates throughout the experiment for unknown reasons and two birds dropped tail feathers during handling (a common antipredator response^99^); importantly, the loss of a tail may influence locomotion^100^. These three birds were therefore excluded from analyses of activity rate. One bird did not produce sperm at three weeks post-inoculation, and was therefore excluded from analyses of sperm count and deformed sperm proportion. In addition, max T differential data for one bird that died during the second sampling session were excluded from analysis. All analyses were performed in R.

### Ethics declarations

This research was carried out in accordance with institutional, state and federal guidelines for animal capture, care, and research. Birds were captured and banded under Federal Bird Banding Permit 20261 and Indiana Scientific Purposes License 20-528. Research was approved under Indiana University Bloomington Institutional Animal Care and Use Committee protocol 18-030-14. This study was carried out in compliance with the ARRIVE guidelines (https://arriveguidelines.org).

### Supplementary Information

Supplementary Information.

## Data Availability

Data are available at https://doi.org/10.5281/zenodo.7992397. The sequence of the inoculating *Plasmodium* strain has been submitted to GenBank (NCBI) as Accession Number OP807039.
