# Supplementary material for: Physiological impacts of chronic and experimental Plasmodium infection on breeding-condition male songbirds
Source: Sci Rep. 2023 Aug 11;13:13091. doi: 10.1038/s41598-023-38438-6 (PMC10421889; doi:10.1038/s41598-023-38438-6)
Supplement: Supplementary file 1 — Supplementary Information. [file 41598_2023_38438_MOESM1_ESM.docx]

**SUPPLEMENT**

*Sexing PCR*

To sex each bird, we ran PCR in 10 μL reactions, which each included: 1 μL undiluted DNA, 1.5 μL each of primers P2 and P8 (Griffiths et al. 1998 *Molecular Ecology*) at 10 μM, 2 μL nuclease-free water, 1 μL dNTPs at 2 mM, 2 μL GoTaq Green Flexi Buffer (Promega Corporation), 0.8 μL MgCl_2_ at 25 mM, and 0.2 μL GoTaq. The conditions were as follows: a denaturing step of 1 minute at 95°C, followed by 30 cycles of 95°C for 20 sec, 53°C for 30 sec, and 72°C for 30 sec. PCR products were visualized after running electrophoresis at 90 v for 1.5 hrs on a 1.5% agarose gel stained with GelRed (Biotium).

*Nested PCR for parasite sequencing*

To isolate parasite DNA for sequencing potential donors, we used a nested PCR approach modified from Hellgren et al. 2004 *Journal of Parasitology*. For the first round of PCR, we ran 15 μL reactions, which each included: 1.5 μL each of primers HaemNFI and HaemNR3 at 10 uM, 2 μL of undiluted DNA, 2.5 μL of nuclease-free water, and 7.5 μL of commercial PCR master mix (Promega). For the second round of PCR, we ran 15 μL reactions, which each included: 1.5 μL each of primers HaemF and HaemR2 at 10 uM, 2 μL of round one PCR product as template, 2.5 μL of nuclease-free water, and 7.5 μL of commercial PCR master mix (Promega). Both rounds were run using conditions described in Hellgren et al. 2004, *Journal of Parasitology*. PCR products were visualized after running electrophoresis at 90 v for 45 min on a 1% agarose gel stained with GelRed (Biotium).

*Single step PCR for diagnosis of chronic* Plasmodium *infections*

To diagnose chronic *Plasmodium* infections, we ran PCR in 10 μL reactions. Each reaction included: 1 μL each of primers L9 and NewR (Knowles et al. 2010 *Journal of Evolutionary Biology*) at 4 μM, 3 μL of DNA diluted to 2 ng/μL, and 5 μL of commercial PCR master mix (Promega). Conditions for PCR were as follows: an initial denaturation step at 95°C for 2 min, followed by 43 cycles of 95°C for 15 sec, 60°C for 30 sec, and 72°C for 30 sec. PCR products were visualized after running electrophoresis at 90 v for 45 min on a 1% agarose gel stained with GelRed (Biotium). Samples showing no amplification were screened a second time to reduce the likelihood of false negatives. Samples showing no amplification after two runs were scored as uninfected.

Figure S1. Experimental timeline from June 12 – July 30, 2020. Baseline and post-inoculation sampling sessions included collection of the following data: activity rate (movements/min), body condition index, hematocrit, and cloacal protuberance volume (mm^3^). These data were collected twice during the baseline period, and once per week during post-inoculation sessions. Inoculations and sampling occurred in three-day rotations, with one room of birds sampled per day; the order of room sampling was held constant across the project to ensure all birds were sampled exactly once per week.

Figure S2. GnRH-induced testosterone concentrations in breeding-condition male juncos prior to and following experimental inoculation with *Plasmodium*. A) Pre-inoculation testosterone in males with (‘1’) and without (‘0’) chronic *Plasmodium* infections; B) testosterone differentials (post-inoculation minus pre-inoculation values) in *Plasmodium*-inoculated (‘1’) and control (‘0’) males. Dots represent individual birds. Values reflect non-transformed data.
